# Evaluation of an approach to clinical decision support for preventing inpatient falls: a pragmatic trial

**DOI:** 10.1093/jamiaopen/ooad019

**Published:** 2023-04-06

**Authors:** Insook Cho, MiSoon Kim, Mi Ra Song, Patricia C Dykes

**Affiliations:** Nursing Department, Inha University, Incheon, Republic of Korea; Center for Patient Safety, Research, and Practice, Brigham and Women’s Hospital, Boston, Massachusetts, USA; Department of Nursing, Samsung Medical Center, Seoul, Republic of Korea; Department of Nursing, Samsung Medical Center, Seoul, Republic of Korea; Center for Patient Safety, Research, and Practice, Brigham and Women’s Hospital, Boston, Massachusetts, USA; Department of Medicine, Harvard Medical School, Boston, Massachusetts, USA

**Keywords:** inpatient falls, clinical decision support, machine learning, prediction model, clinical evaluation, tailored interventions

## Abstract

**Objectives:**

To assess whether a fall-prevention clinical decision support (CDS) approach using electronic analytics that stimulates risk-targeted interventions is associated with reduced rates of falls and injurious falls.

**Materials and Methods:**

The CDS intervention included a machine-learning prediction algorithm, individual risk-factor identification, and guideline-based prevention recommendations. After a 5-month plan-do-study-act quality improvement initiative, the CDS intervention was implemented at an academic tertiary hospital and compared with the usual care using a pretest (lasting 24 months and involving 23 498 patients) and posttest (lasting 13 months and involving 17 341 patients) design in six nursing units. Primary and secondary outcomes were the rates of falls and injurious falls per 1000 hospital days, respectively. Outcome measurements were tested using a priori Poisson regression and adjusted with patient-level covariates. Subgroup analyses were conducted according to age.

**Results:**

The age distribution, sex, hospital and unit lengths of stay, number of secondary diagnoses, fall history, condition at admission, and overall fall rate per 1000 hospital days did not differ significantly between the intervention and control periods before (1.88 vs 2.05, respectively, *P *=* *.1764) or after adjusting for demographics. The injurious-falls rate per 1000 hospital days decreased significantly before (0.68 vs 0.45, *P *=* *.0171) and after (rate difference = –0.64, *P *=* *.0212) adjusting for demographics. The differences in injury rates were greater among patients aged at least 65 years.

**Conclusions:**

This study suggests that a well-designed CDS intervention employing electronic analytics was associated with a decrease in fall-related injuries. The benefits from this intervention were greater in elderly patients aged at least 65 years.

**Trial Registration:**

This study was conducted as part of a more extensive study registered with the Clinical Research Information Service (CRIS) (KCT0005378).

## INTRODUCTION

Inpatient falls are frequent adverse events that are responsible for various injuries experienced by approximately one-third of hospitalized patients.^1^^,^^2^ The South Korean national system for patient safety reported that more than 4800 inpatient falls with any injury were registered from August 2016 to December 2019, among which 1480 were severe injuries such as fractures and head injuries.^3^ That research team found that the incident reporting system of one tertiary hospital indicated that 25% of patients experienced injurious falls, while the injury rate was 36.9% at another hospital.^4^

The US Centers for Medicare and Medicaid Services stopped care reimbursements for inpatient fall-related injuries in 2008.^5^ The South Korean government introduced a requirement for active inpatient fall-prevention programs as a criterion for hospital accreditation in 2018.^6^ The enactment of the Patient Safety Act in 2016 in South Korea has further increased the interest and concerns in hospitals toward addressing inpatient falls.

International practice guidelines, including those from the UK National Institute for Health and Care Excellence (NICE), the Registered Nurses Association of Ontario of Canada (RNAO), and a toolkit developed by the US Agency for Healthcare Research and Quality (AHRQ), unanimously recommend conducting multifaceted risk assessments and identifying individual fall risk factors in hospitalized patients.^7^ Risk-targeted interventions should then be followed by implementing multifactorial activities such as patient and caregiver engagement, communication among caregivers, and interventions tailored to patient-specific risk factors such as providing assistance in getting out of bed, toileting schedules, and alarm-device use. Several studies^8–11^ have explored the effectiveness of these guideline recommendations using randomized clinical trials (RCTs), but their results have been mixed. One US research team reported using a fall-prevention tool kit (FPTK) that included clinical decision support (CDS) for individually tailored interventions that reduced fall rates, particularly for patients aged 65 years or older.^10^ In another clinical trial performed by the same study team,^11^ the FPTK intervention contributed to 15% and 34% reductions in falls and injurious falls, respectively, with the decreases being greatest among patients younger than 65 years and injury reductions being most significant in patients aged 65 years or older. Two Australian RCTs^8^^,^^9^ that applied targeted multifactorial prevention programs that were not tailored to patient-specific risk factors found no significant effects.

Electronic algorithms that forecast clinical events, called electronic healthcare predictive analytics, are increasingly being used to prevent poor clinical outcomes.^12^ In recent years, several inpatient fall prediction models have been published in the healthcare literature, but their clinical impacts on patient outcomes remain to be demonstrated. Our research team developed a fall-risk prediction model based on electronic nursing data and tested its clinical feasibility and user acceptance in previous studies.^4^^,^^13^ Before moving to expensive large-scale trials, it is necessary to accurately evaluate the performance of the prediction model and coordinate its practice workflow. This study aimed to assess whether the fall-prevention CDS approach using machine learning that stimulates risk-targeted interventions is associated with reductions in the rates of falls and injurious falls among hospital inpatients.

## BACKGROUND

**Figure 1.**
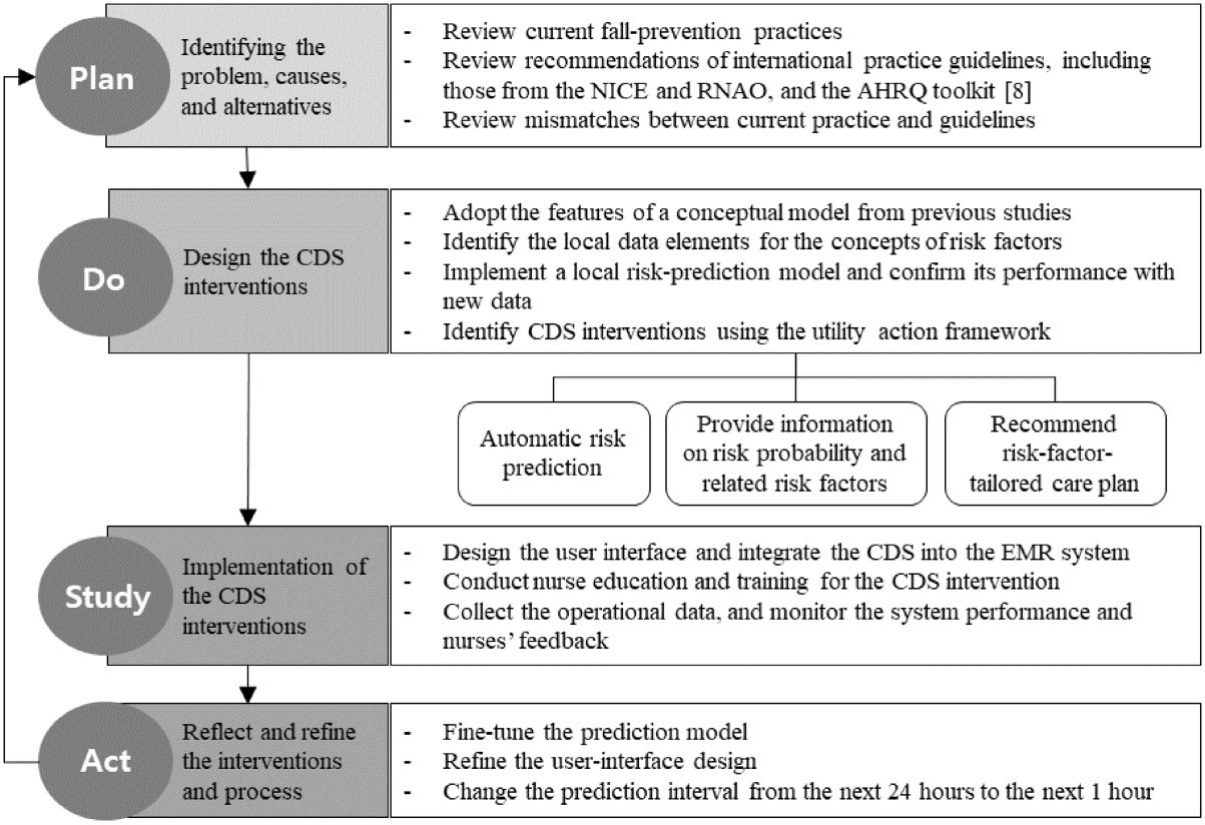
Overview of the intervention development process that used a systematic framework to develop and apply the electronic healthcare predictive analytics^19^ and utility action framework.^21^ AHRQ: US Agency for Healthcare Research and Quality; NICE: UK National Institute for Health and Care Excellence; RNAO: Registered Nurses Association of Ontario of Canada; CDS: clinical decision support; EMR: electronic medical records.

The CDS intervention was developed iteratively using multiple steps based on the plan-do-study-act (PDSA) model for quality-improvement research.^14^^,^^15^ The process is depicted in Figure 1. Each stage included several activities to improve the fall prevention process, develop and introduce the CDS approach, and carry out the required interventions. In stage 1 of the plan, we assembled a fall prevention team consisting of nurse managers and leadership, unit champions, staff nurses, nursing informaticians, and personnel from the quality department and the department of health information technology. Academic channels were also established for facilitating collaborations. We first examined the variability of patient outcomes and the current practice for risk assessments and preventive interventions using the hospital’s electronic medical records (EMR) data. We then identified the baseline frequencies and compliance rates of risk-targeted interventions, which were recommended by international practice guidelines including those from the NICE and RNAO, and the AHRQ toolkit. We found mismatches between the risk factors identified and the preventive interventions provided to each patient, which led us to describe the problems and what interventions needed to be implemented. The team focused on helping nurses to conduct evidence-based fall-risk assessments with a lower cognitive burden, which could guide or remind them about the appropriate subsequent activities.

In stage 2, the research team adopted a conceptual model of inpatient fall-risk prediction developed using clinical concepts derived from seven US and one South Korean fall-prevention guideline.^4^ The conceptual model was used to realize the hospital’s local model by mapping data elements to the hospital’s EMR system. We used 31 937 admissions (284 667 hospital days) from 9 nursing units covering the period from January 1, 2017 to June 31, 2018. We conducted rule-based automatic free-text reviews of clinical notes to identify unreported fall events and included all clear cases of fall events. After comparing the performance of logistic regression, artificial neural network, random forest, and gradient boost algorithms, the final model was implemented using the eXtreme Gradient Boosting (XGB) algorithm. This algorithm performed better than other healthcare algorithms,^16^^,^^17^ in comparisons that referenced a systematic framework suggested by Amarasingham et al^18^ for guiding the development and application of electronic healthcare predictive analytics. According to the five recommendations of the framework, we set model validation criteria based on the performance on the Morse Fall Scale (MFS) and simulated the model’s performance. The performance of the final model was assessed using 26 months data from nursing units, which revealed an area under the receiver operating characteristic curve of 0.85.

The XGB model was integrated into the EMR system. The following CDS functions were designed: providing patient-level next-hour prediction results, updating related risk factors hourly, and populating specific nursing activities tailored to individual risk factors. The prediction results were reported in the form of probabilities. Using the feature importance calculated from feature ranking with an information-gain algorithm and sensitivity analyses, the research team set a threshold probability of 15% for when a patient was judged as being at risk. Nurses were notified of at-risk patients with a fall risk higher than this threshold along with specific risk factors in six categories: cognition (dementia or delirium, or lack of cooperation), toileting, mobility, culprit medication, sensory status, and sleep. These six categories for preventing inpatient falls were based on a Korean guideline.^19^ The at-risk classification triggered populating a tailored care plan based on specific risk factors. The standard activities of the care plan were adopted from the standardized terminology-based inpatient-fall-prevention catalog informed by evidence, a theoretical framework, and clinical validation.^6^

This CDS function design process followed the utility action framework suggested by Liu et al.^20^ Considering the critical action elements, the unit-level capacity and number of patients who would benefit from the intervention were considered when setting the at-risk threshold, and the risk predictions were integrated into the existing workflow of nurses. To obtain user feedback, the nurses were allowed to override the risk-prediction results and enter their reasons on the EMR screen. Based on end-user feedback on the prediction model,^21^ we provided a screen to illustrate how and from which data a risk probability was generated.

The next study stage comprised the implementation of the CDS system and its integration with the EMR system. We constructed an algorithm monitoring team to supervise its performance and provide education and training sessions to nurses. The CDS functions were delivered to four nursing units, and monitoring was carried out after their release, thereby providing more opportunities to identify problems. We collected and investigated data on the model, nurses’ feedback, and management or workflow issues every week from April 1 to August 31, 2020. The data analysis revealed that the number of falls had decreased and that there were no data errors. The prediction results were acceptable to nurses who did not report any significant workflow issues. Nurses felt that they were greatly supported by the automatic risk predictions and reminders on risk factors for each patient. In a few cases, nurses changed a patient from a no-risk to an at-risk status. The reasons for nurses overriding risk-prediction results were related to their intuition, such as the body strength of a patient appearing to have decreased, which was not yet captured in the data. Another issue was the time of diagnostic tests or procedures using sedative or anesthetic medications being scheduled but not yet implemented when the algorithm was executed. Six fall cases that had been predicted as no fall risk were due to a lack of available data in the EMR system.

In the act stage of the study, the research team fine-tuned the prediction model. The user interface design displaying each patient’s fall probability and risk factors was refined according to nurses’ feedback about where on the screen information was displayed and the font size used. Several risk-targeted prevention activities were found to be populated redundantly across risk categories, and so they were reorganized. We also changed the prediction interval from the next 24 h to the next 1 h to reflect the availability of real-time data. Based on the iterative processes and CDS refinement, the overall satisfaction of nurses with the CDS interventions increased by > 80% in the hospital’s quality-improvement survey. The CDS intervention was ready for release in the next PDSA cycle, focusing on its effects on patient outcomes.

## METHODS

### Study design

A pragmatic pretest–posttest design was used to test the fall-prevention CDS intervention. This study was conducted at an academic tertiary hospital in Seoul. The following six nursing units were selected (which included the four units in which the pilot study had been performed): a transplant surgery unit, a rehabilitation unit, two respiratory internal medicine units, and two hematology units. These units all had fall rates that were higher than the mean rate across the hospital. The study team provided the introduction and training sessions of the CDS intervention to the units’ nurses. The six units all met the participation criteria: inpatients aged 18 years or older, not associated with psychiatry or obstetrics, and availability of 24 months of preintervention data. The study was approved by the hospital’s institutional review board (IRB) (approval no. SMC202005005003). Due to the quality-improvement nature of the CDS intervention, the IRB waived the requirement to obtained informed consents from the participants. The study followed the TREND (Transparent Reporting of Evaluations with Nonrandomized Designs) reporting guidelines.^22^

### Fall-prevention CDS intervention

We developed a CDS with fall-prevention predictive algorithm. The fall-risk prediction results for each patient are presented to nurses on EMR screens. The MFS was used only on the admission day, and the CDS intervention was provided during the experimental period. Nurses were guided in interpreting the prediction result for each patient and recording a care plan. The CDS intervention was integrated with the EMR system, which allowed all nurses to check the prediction results at least once during a work shift or whenever the condition of a patient changed.

For the control period, nurses used the built-in modified MFS to assess fall risks which was the usual care process at the hospital. The care process includes a 45-point cutoff score, with patients above this level judged as being at-risk; nurses would provide vigorous preventive activities to such patients based on their judgment. An educational program on fall-risk assessment and prevention was provided as the standard during both study periods. Table 1 compares the fall-prevention protocols used during the control and experimental periods.

**Table 1.** Fall-prevention protocols during the control and experimental periods

	Control period	Experimental period
	(usual care)	(CDS intervention)
Fall-risk assessment (daily and following a status change)	MFS and three additional items	Electronic fall-risk prediction algorithm
Fall-risk information on EMR screen	No risk information. Binary result of at-risk or not at-risk	Binary result of at-risk or not at-risk, along with the fall probability and risk factors
Fall-prevention plan documentation	Not supported	Recommend list of tailored plans generated by CDS function based on risk factors

*Abbreviations*: CDS: clinical decision support; EMR: electronic medical records; MFS: Morse Fall Scale.

### Outcomes

The primary outcome was the number of patient falls per 1000 hospital days in the six units. A secondary outcome was the number of fall-related injuries per 1000 hospital days during the control and experimental periods. The outcome data during the control period were already routinely collected by the event reporting system of the hospital. During the experimental period, data on falls and the resulting injury levels were also routinely recorded in an event reporting system by the clinician caring for the patient at the time of their fall. To validate and identify unreported events during both periods, all text inputs of nursing notes were filtered using a rule-based event detection algorithm, and quality-improvement personnel at the hospital reviewed and judged suspicious events. These data were abstracted and entered into the annotated control chart for analysis, which was available only to data analysts.

### Statistical analysis

This study used a cluster design with pre- and postintervention periods in six units. Falls were measured at the patient level. Patients were randomly allocated to nursing units by the hospital admissions department. Differences in the rate of falls between the pre and postintervention periods were examined using an a priori Poisson regression model containing a time effect. The monthly numbers of falls according to unit and total hospital days as an offset variable were used. We also tested for any residual effect of clustering within a unit using a generalized estimating equation method. In adjusted Poisson regression tests, the following variables related to falls were controlled: age, sex, fall history, and the number of secondary medical diagnoses.

For the subgroup analyses, we assessed whether the changes before and after the intervention differed between those younger than 65 years and those aged at least 65 years. We also examined differences among the units according to period using the adjusted Poisson regression models. Patient characteristics in the two periods were summarized using proportions for categorical variables and means with standard deviations (SDs) for continuous variables. The covariate balance for the patient characteristics during the two periods was analyzed using standardized differences.^23^ The reported *P* values are all two-sided, and statistical significance was set at *P *<* *.05. We used SAS software (version 9.4, SAS Institute) for the statistical analyses.

## RESULTS

This study analyzed 40 839 admissions that corresponded to 263 484 hospital days. There were 23 498 and 17 341 admissions during the pre- and postintervention periods, respectively (Table 2). The following parameters did not differ significantly between these two periods: age distribution, sex, hospital and unit lengths of stay, secondary diagnoses, fall history, or conditions at admission, such as dementia, need for aids, or sleep disturbance. The ages at admission were 58.97 ± 13.85 and 59.27 ± 14.02 years (mean ± SD) before and after the intervention, respectively. There were 13 458 (57.3%) and 9899 (57.1%) male patients, before and after the intervention, respectively. The hospital and unit lengths of stay were 10.28 ± 19.57 and 6.56 ± 8.88 days, respectively, before the intervention and 10.77 ± 40.44 and 6.65 ± 8.42 days after the intervention. All standardized differences comparing demographics and admission statuses across periods were <10%, suggesting that the covariates were well-balanced between the periods^23^^,^^24^ (Table 2). The variables of age, sex, unit length of stay, fall history, and secondary diagnoses were used to adjust the a priori Poisson regression analyses.

**Table 2.** Admission characteristics and standardized differences between before and after implementation of the fall-prevention CDS intervention

Characteristics	Before the intervention	After the intervention	Standardized difference^a^ (%)
Patients	23 498	17 341	NA
Hospital-days	147 805	115 679	NA
Age, years	58.97 ± 13.85	59.27 ± 14.02	−2.12
Sex, male	13 458 (57.3)	9899 (57.1)	−0.40
Hospital length of stay, days	10.28 ± 19.57	10.77 ± 30.44	−1.93
Unit length of stay, days	6.56 ± 8.88	6.65 ± 8.42	−0.99
No. of secondary diagnoses	0.94 ± 0.23	0.95 ± 0.22	−3.08
Fall history at admission	1457 (6.2)	794 (4.6)	−7.08
Dementia diagnosis at admission	230 (1.0)	151 (0.9)	−1.03
Need for aids at admission	2772 (11.8)	1898 (10.9)	−2.84
Sleep pattern disturbance at admission	1995 (8.5)	1.420 (8.2)	−1.08

*Note*: Data are mean ± SD, *n*, or *n* (%) values.

a Standardized differences with absolute values of <10% reflect covariates that are well-balanced across periods.

The adherence of nurses to the intervention protocol was estimated at 100% for both periods because nurses are required to assess and document fall precautions at least once a day during their work shifts. During the intervention, nurses had to observe the risk-prediction results in order to be able to advance to the documentation screen. We randomly checked the compliance and documentation for fall prevention, which did not identify any missing records.

In the outcome analysis, the overall fall rate per 1000 hospital days did not differ significantly between the intervention period (1.88; 95% confidence interval [CI], 1.58–2.19) and the control period (2.05; 95% CI, 1.80–2.30) (Table 3). The injury rates per 1000 hospital days decreased significantly from 0.68 (95% CI, 0.55–0.81) during the control period to 0.45 (95% CI, 0.31–0.58) during the intervention period (*P *=* *.0171). After adjusting for covariates using the Poisson regression model, the overall fall-rate decrease of −0.08 (95% CI, −0.328 to 0.16) was not significant. However, after adjusting for covariates, the −0.64 change in the overall injury rate (95% CI, −1.18 to −0.10) was significant (*P* = .0212). There was a significant interaction between age group and period (*P *=* *.0253).

**Table 3.** Participant falls and adjusted fall rates before and after the intervention

	Before the intervention	After the intervention	Rate difference	*P*
All patients				
No. patients with falls/total no. of patients	254/23 498	185/17 341	–	–
Total no. of falls	295	203	–	–
No. of repeat falls	19	9	–	–
Fall rate (95% CI) per 1000 hospital days	2.05 (1.80–2.30)	1.88 (1.58–2.19)	−0.17 (−0.22 to 0.55)	.1764
Fall rate (95% CI) per 1000 hospital days adjusted for age, sex, unit, fall history, and no. of secondary diagnoses	2.01 (1.68–2.42)	1.85 (1.52–2.26)	−0.08 (−0.32 to 0.16)	.5019
No. of falls with injury	77	52		
Injury rate (95% CI) per 1000 hospital-days	0.68 (0.55–0.81)	0.45 (0.31–0.58)	−0.23 (−0.42 to 0.04)	.0171
Injury rate (95% CI) per 1000 hospital days adjusted for age, sex, unit, fall history, and no. of secondary diagnoses	0.72 (0.54–0.96)	0.38 (0.28–0.51)	−0.64 (−1.18 to −0.10)	.0212
Patients aged <65 years				
Total no. of falls	148	84		
Fall rate (95% CI) per 1000 hospital-days	1.03 (0.83–1.22)	1.08 (0.85–1.31)	0.05 (−0.27 to 0.36)	.3725
Fall rate (95% CI) per 1000 hospital days adjusted for sex, unit, fall history, and no. of secondary diagnoses	1.54 (1.21–1.94)	1.77 (1.62–1.94)	0.14 (−0.16 to 0.45)	.3659
No. of falls with injury	40	20		
Injury rate (95% CI) per 1000 hospital-days	0.33 (0.23–0.43)	0.26 (0.14–0.37)	−0.07 (−0.23 to 0.08)	.1718
Injury rate (95% CI) per 1000 hospital days adjusted for sex, unit, fall history, and no. of secondary diagnoses	0.52 (0.38–0.72)	0.34 (0.24–0.50)	−0.41 (−1.08 to 0.26)	.2285
Patients aged ≥65 years				
Total no. of falls	147	63		
Fall rate (95% CI) per 1000 hospital-days	1.02 (0.84–1.20)	0.81 (0.60–1.02)	−0.21 (−0.50 to 0.08)	.3357
Fall rate (95% CI) per 1000 hospital days adjusted for sex, unit, fall history, and no. of secondary diagnoses	2.59 (2.08–3.22)	1.75 (1.08–2.83)	−0.39 (−0.99 to 0.21)	.1974
No. of falls with injury	50	15		
Injury rate (95% CI) per 1000 hospital-days	0.35 (0.25–0.44)	0.19 (0.10–0.28)	−0.16 (−0.30 to −0.01)	.0220
Injury rate (95% CI) per 1000 hospital days adjusted for sex, unit, fall history, and no. of secondary diagnoses	0.92 (0.70–1.20)	0.33 (0.22–0.49)	−1.03 (−1.60 to −0.46)	.0004

CI: confidence interval.

In the subgroup analysis by age, the decreases in fall and injury rates were larger for older (≥65 years) than younger (<65 years) patients. For younger patients, the differences in fall and injury rates were not significant in both the unadjusted and adjusted analyses. In older patients, the fall-rate differences were not significant. Meanwhile, the differences in injury rates were greater than those in overall injury rates, with significance in both the unadjusted (−0.16; 95% CI, −0.30 to −0.01; *P *=* *.0220) and adjusted (−1.03; 95% CI, −1.60 to −0.46; *P *=* *.0004) analyses.

**Figure 2.**
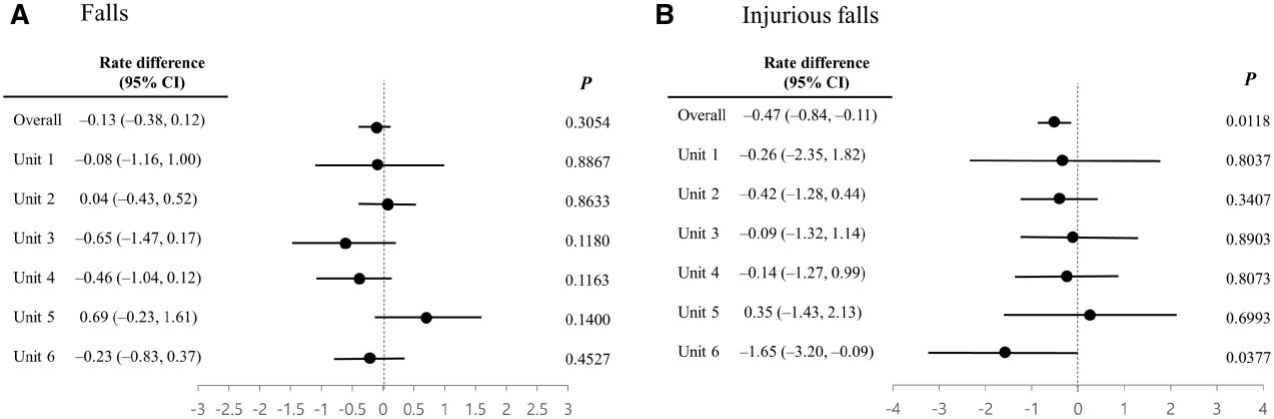
Adjusted differences in the rates of falls and injurious falls according to unit before and after intervention. CI: confidence interval.

Figure 2 shows the adjusted differences in fall and injury rates per 1000 hospital days according to the nursing unit. The response pattern at the rehabilitation unit differed from those at the other units for both outcomes.

## DISCUSSION

This study evaluated a clinical guideline-driven and systemic fall-prevention approach using a machine-learning technique in a large academic tertiary hospital. The fall rate decreased after the intervention was not supported statistically. However, the rate of injurious falls reduced significantly after the intervention, and this reduction remained significant after adjusting relevant patient-level characteristics. The fall-prevention CDS intervention was more significantly associated with decreasing injurious falls for patients aged 65 and older. Following previous studies,^4^^,^^13^ the present trial demonstrated that integrating machine-learning predictions and guideline-driven CDS functions into the EMR could help to reduce the considerable physical and cognitive burdens experienced by nurses when they are ensuring patient safety. To improve the effectiveness of this intervention further, combining already-known effective strategies might help to decrease fall rates, such as routine patient and family engagement and other quality improvement tools.

This was the first fall-prevention clinical study to provide evidence for combining a machine-learning technique and CDS functions for EMR systems. A previous evaluation^13^ of the feasibility of fall-risk prediction using machine learning found that changes in nurse behavior resulted in significant improvements in risk-targeted, preventive activities. In the current study, we added more-sophisticated CDS functions to the EMR system to remind nurses about guideline-driven activities tailored to patient-level risk factors. Regarding efforts to predict the inpatient fall-risk status using EMR data, several studies have focused on building models or comparing methods using various machine-learning techniques^16^^,^^25^^,^^26^ or logistic regression.^27^ However, most of these approaches were not validated prospectively in other settings or clinical practice.

It is essential to engage and communicate with clinicians, other hospital staff, and patient families about risk factors for preventing inpatient falls. Dykes et al^11^ developed the Fall Tailoring Interventions for Patient Safety (TIPS) tool kit. They used bedside tools to communicate patient-specific fall risk factors and a tailored prevention plan. The Fall TIPS consist of three components: (1) assessing the fall risk, (2) using CDS to link patient-specific risk factors to tailored interventions, and (3) executing intervention plans consistently. These three components of the Fall TIPS were similar to those in the current study, but different implementation modalities were applied since we used machine learning to assess the fall risk. Both approaches use the CDS functions of an EMR system to produce personalized prevention plans. Our approach was more focused on nurses and the nursing process, while the Fall TIPS were weighted toward patient engagement. Significant fall-rate reductions were found in an early Fall TIPS study, particularly among patients aged 65 years or older, with no decrease in injurious falls among four urban US hospitals.^10^

Performance feedback and benchmarking are standard methods used for improving quality. In the current study, we observed that some nurses found the new intervention burdensome or doubted its benefits because they were unfamiliar with it and were not informed about it or encouraged to use it. Kiefe et al^28^ demonstrated that using achievable benchmarks significantly enhanced the effectiveness of physician performance feedback in quality-improvement interventions. Achievable benchmarks are data-driven rather than being based on traditional and subjective opinions. Bourdeaux et al^29^ examined the impact of behavioral changes on clinical decision-making to increase physician compliance in intensive-care units. One intervention was using a dashboard, which rapidly led to considerable improvements in practice. This approach could be used to further enhance the effects of the intervention developed in the present study. The database of the fall-prevention CDS system could be used to efficiently design and implement a real-time dashboard or a performance feedback tool at the unit or patient level.

This study has several considerations when attempting to generalize the results. First, the fall rates were not significantly reduced. This was possibly led by the difference between retrospective tracking and prospective monitoring of fall incidents which originated from the study design. Because non-injurious falls tend not to be reported and recorded compared to injurious falls. In addition, during the intervention period, research staff conducted unit rounding to monitor fall incidents and observe workflow, which might make it hard to ignore and un-report non-injurious falls. Otherwise, the intervention period of 13 months might have been too short to reveal the effect of the intervention on patient falls. Further intervention effects might be revealed when larger numbers of falls and longer hospital days are included. Second, the study was conducted at a single large tertiary hospital in an urban area. The patient population was younger than in other acute-care settings, which we attributed to the primary diagnosis being neoplasm in >40% of the patients. In our previous study setting,^13^ the average age of patients was 61.5–65.3 years, and respiratory and gastrointestinal diseases were the most common. The inclusion of a younger population might help to hide the intervention effects that contribute to injuries in older patients. Further research is required in settings with older patient populations with a higher prevalence of cognitive impairment and multicomplexity, including polypharmacy, which may reveal even stronger effects of the CDS intervention. Third, the six clustered nursing units involved in this study were heterogeneous compared with those in other evaluation studies. Specifically, the response patterns to the intervention at the rehabilitation unit differed from those at the other medical–surgical units. This might have been related to the patients being older, having a 2.5-times longer stay, and being more likely to have mobility problems as risk factors compared with the other medical–surgical units. These different characteristics may need more-specific prediction algorithms or intervention strategies, which should be explored in future work.

One of the main strengths of this study was that it revealed that the CDS intervention was associated with a reduction of injurious falls. This implies the possibility that the three main features of the CDS intervention have stimulated nurses to do more sophisticated practice than in the pretest period. We have observed the practice changes in our previous study.^13^ The reduction effects of fall-related injuries for patients aged 65 years or older was a meaningful result considering that these injuries are strongly associated with increased care costs and undesirable patient outcomes. The present results indicate that the CDS intervention can prevent 1 fall-related injury per 4348 hospital days, which corresponds to 621 patients being treated over the typical 7-day stay. There were on average 28 events experienced by 621 patients each year in the six study units. The CDS intervention could therefore prevent 28 fall injuries yearly in the studied units.

## CONCLUSION

The CDS intervention with the predictive algorithm was associated with a reduction in injurious fall rates, with a larger reduction in patients aged 65 years or older. However, the intervention was not associated with a significant reduction in fall rates. Additional strategies for implementing guideline recommendations—such as patient and family engagement and using achievable benchmarks or unit-level feedback combined with machine-learning techniques—should be explored further with the aim of increasing the efficacy of the intervention.

## Data Availability

The data underlying this article were provided by Samsung Medical Center in Seoul by permission. Data will be shared on request to the corresponding author with permission of the Samsung Medical Center.
